# Two-Year Preclinical Evaluation of Long-Term Absorbable Poly-4-hydroxybutyrate Scaffold for Surgical Correction of Pelvic Organ Prolapse

**DOI:** 10.1007/s00192-023-05720-0

**Published:** 2024-03-02

**Authors:** Zeliha Guler, Lisa Ann Kaestner, Eva Vodegel, Lamees Ras, Stephen Jeffrey, Jan Paul Roovers

**Affiliations:** 1 Department of Obstetrics and Gynaecology, Amsterdam UMC – location AMC, University of Amsterdam, Meibergdreef 9, Amsterdam, The Netherlands; 2 Amsterdam Reproduction and Development, Amsterdam, The Netherlands; 3 Department of Urology, Groote Schuur Hospital, University of Cape Town, Cape Town, South Africa; 4 Department of Obstetrics and Gynecology, Groote Schuur Hospital, University of Cape Town, Cape Town, South Africa

**Keywords:** Pelvic organ prolapse, Vaginal surgery, Poly-4-hydroxybutyrate, Absorbable scaffold, Long-term host response, Biomechanics

## Abstract

**Introduction and hypothesis:**

Fully absorbable implants may be an alternative to permanent meshes in the correction pf pelvic organ prolapse (POP) as they may reduce adverse events by promoting tissue regeneration and collagen metabolism. This study was aimed at evaluating the long-term host and biomechanical response to a fully absorbable poly-4-hydroxybutyrate (P4HB) scaffold in comparison with polypropylene (PP) mesh.

**Methods:**

Poly-4-hydroxybutyrate scaffold (*n* = 16) and PP mesh (*n* = 16) were surgically implanted in the posterior vaginal wall of parous female Dohne Merino sheep. Vaginal explants were evaluated in terms of gross necropsy, host response (immune response, collagen deposition, tissue regeneration), biomechanics, and degradation of P4HB at 12 and 24 months post-implantation.

**Results:**

Gross necropsy revealed no infection or fluid collection using P4HB or PP. At 12 months, exposures were observed with both P4HB (3 out of 8) and PP (4 out of 8), whereas at 24 months, exposures were observed only with PP (4 out of 8). The tensile stiffness of the P4HB explants was maintained over time despite complete absorption of P4HB. The collagen amount of the vaginal tissue after P4HB implantation increased over time and was significantly higher than PP at 24 months. P4HB scaffolds exhibited significantly lower myofibroblast differentiation than PP meshes at 24 months.

**Conclusions:**

The P4HB scaffold allowed for gradual load transfer to the vaginal wall and resulted in mechanically self-sufficient tissue. P4HB scaffold had a more favorable host response than PP mesh, with higher collagen content, lower myofibroblastic differentiation, and no exposures at 24 months. P4HB scaffolds have potential as an alternative to permanent implants in treating POP.

**Supplementary information:**

The online version contains supplementary material available at 10.1007/s00192-023-05720-0.

## Introduction

Pelvic organ prolapse (POP) is a multifactorial disorder caused by damage to the supportive structures of the pelvic floor [1, 2] that results in the descent of pelvic organs into the vagina. It is a prevalent disorder with an incidence of 1 out of 4 women, and 40–60% of parous women over the age of 50 years [3]. The pathophysiology of POP is still not entirely understood; however, there is evidence that disturbed fibroblast function impairs collagen metabolism and affects the mechanical properties of pelvic tissues [2, 4, 5]. Native tissue repair (NTR) is used as a primary surgical procedure for the anatomical correction of POP. However, the functional and anatomical recurrence is high, as a patient’s weakened tissue, as per the definition of inferior quality in women with POP, is used to restore the defect. To reduce the risk of recurrence, polypropylene (PP) meshes have been introduced [6] to provide not only mechanical support but also to induce the formation of new connective tissue via foreign body response [7]. However, initially, PP meshes had high density and stiffness that resulted in excessive scar tissue formation, fibrosis, and eventually adverse events [8]. The FDA released warnings on the use of transvaginal meshes [9] and reclassified transvaginal mesh from class II to class III devices, required post-market surveillance (i.e., 522 studies) to prove the superiority of transvaginal meshes over NTR, and commercial transvaginal meshes were banned in some countries [3, 10–12]. Although PP meshes have evolved to be more tissue compatible, adverse events are predicted owing to their nondegradability [3, 13, 14].

We hypothesize that a delayed absorbable implant would be a viable alternative to PP meshes, as the benefits of an implant are applicable whereas the host response is milder as gradual absorption allows replacement of the functional connective tissue over time. The long-term adverse events can be eliminated or limited if an absorbable implant can maintain its mechanical strength for sufficient time to allow regeneration of mechanically self-sufficient vaginal tissue. Our group has investigated a prototype poly-4-hydroxybutyrate (P4HB) scaffold for the surgical correction of POP. The scaffold triggered the collagen metabolism of vaginal POP fibroblasts in vitro [4] and generated a moderate host response after vaginal implantation into a validated sheep model in the short term [10]. The current study is aimed at evaluating the long-term host and biomechanical response to a fully absorbable prototype P4HB scaffold in comparison with PP mesh in a sheep model that has predictive value in relation to the surgical outcome in humans.

## Materials and Methods

### Setting

This study was conducted at Mariendahl experimental farm, which is a university facility for the Department of Animal Science. Guidelines for the care and use of laboratory animals of the National Health and Medical Research Council of South Africa were followed, including daily monitoring and weekly weighing of sheep. The Animal Ethics Committee of the University of Cape Town approved the experimental protocols (AEC A18-036) for the maintenance and treatment of the sheep. Only sheep were used in the study; patients were not involved.

### Implants

**Fig. 1:**
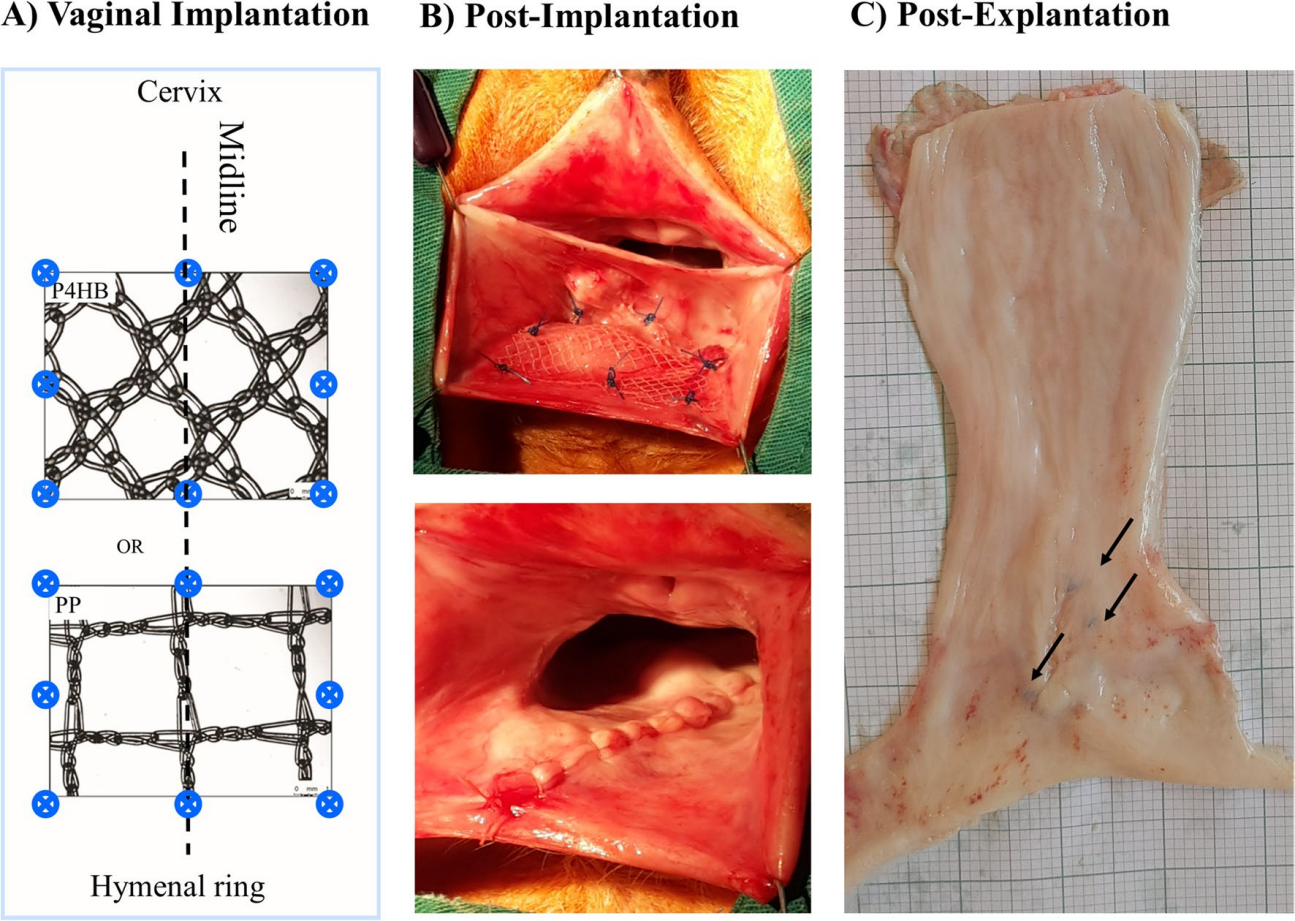
**A** Schematic and **B** photographic representation of poly-4-hydroxybutyrate (*P4HB*) or polypropylene (*PP*) vaginal implantation in the posterior compartment. Fixation points using nonresorbable PP 3/0 sutures and vaginal closure using a running suture with 3/0 polyglactin 910 (Vicryl). **C** After en bloc excision of the vagina, the mucosal side of the vagina shows the healed vaginal closure. *Arrows* indicate the sutures used for the implantation

A prototype monofilament P4HB scaffold (sterile, noncommercial; thickness: 0.28 mm, fiber diameter: 100 µm, pore size: 2.22 mm^2^ [4, 10]) was compared with lightweight PP–Restorelle® (Coloplast, Minneapolis, MN, USA; thickness: 0.34 mm, fiber diameter: 80 µm, pore size: 3.1 mm^2^; Fig. 1A.

### Animals, Surgical Procedures, and Study Design

Thirty-two parous female Dohne Merino sheep (7 years old, weighing 51.5 ± 5.7 kg) were included.

As a larger animal model, sheep can be used for the short- and long-term evaluation of novel implants and it is possible to reproduce vaginal exposures [15, 16]. Although sheep are quadrupeds, the gross and microscopic anatomies are similar to the human female pelvis. The dimensions and morphology of the sheep vagina are comparable with those of the human. The retropubic and the rectovaginal space are accessible transvaginally. There is a wide expression of estrogen receptors in pelvic tissues as in women [17]. Not only the dimensions and anatomy of the ovine vagina and pelvic floor are comparable with those in humans but also the spontaneous occurrence of prolapse, which occurs in 15% of ewes. Prolapse risk factors are overlapping (i.e., multiparity, previous history of POP, increased intra-abdominal pressure induced by a greater body weight or when grazing on hills [18].

Thirty-two sheep were randomly divided into four groups (P4HB and PP, 12 and 24 months) (*n* = 8 per group, see Supplementary material for sample size calculation). All sheep underwent surgery for rectovaginal implantation of P4HB (*n* = 16) or PP (*n* = 16). The surgical procedure was carried out according to the previously described method [10, 19]. P4HB or PP meshes (35 × 35 mm) were fixed with nondegradable 3/0 PP sutures (Prolene®, Ethicon) at the corners and halfway along each side. The vaginal wall was then closed with a running 3/0 polyglactin 910 (Vicryl) suture. Ewes were euthanized at 12 (P4HB: *n* = 8 and PP: *n* = 8) or 24 months (P4HB: *n* = 8 and PP: *n* = 8) after surgery. For more details, see Supplementary material.

### Harvesting Implants and Macroscopic Evaluation

Sheep were premedicated by insertion of an intravenous catheter into the cephalic vein and 0.3 mg/kg midazolam was administered, followed by 3 mg/kg ketamine IV. Gross anatomical examination of the explanted vagina was performed using the following parameters for implant-related complications: fluid collection, exposure of the implant, synechiae, and signs of infection. Exposure is defined as a mesh that is visible through the vaginal epithelium [20, 21]. Vaginal explants (vaginal tissue/implant complex) were dissected into four pieces for assessing passive biomechanical properties, histomorphology, in vivo degradation (only for P4HB), and SEM images.

### Outcome Measurements

#### Passive Biomechanical Properties

The mechanical properties of the vaginal explants were determined with uniaxial tensiometry by using Instron 5544 (Norwood, MA, USA) with a 200N load cell. Explant biomechanics give a good indication of the success of the implant to restore normal tissue support after surgery. As a control, posterior middle vaginal tissue from the same sheep was used. Samples were cut longitudinally (10 × 30 mm), clamped tension free, and the zero elongation was defined as the clamp-to-clamp distance at preload (0.1 N). The load was applied with an elongation rate of 10 mm/min until failure. The strain was calculated by dividing the elongation by the clamp-to-clamp distance and stress by dividing the force by the cross-sectional area. The stiffness (N/mm) of the specimens was determined with the slope of the stress–strain curve in the comfort zone by using OriginPro2018 software (OriginLab Corporation, Northampton, MA, USA) [10].

#### In Vivo Degradation of the P4HB Scaffold

In order to define the amount of remaining scaffold in the tissue and its structure, in vivo degradation of the P4HB scaffold was determined by molecular weight (Mw) change via gel permeation chromatography (GPC) analysis and by morphological scaffold changes via scanning electron microscopy (SEM) (JEOL JSM6700F) following removal of the vaginal tissue from the explants by tissue digestion with collagenase [10] to define the amount of remaining implant in the tissue over time. See Supplementary material 4 for details.

#### Histomorphology

Tissue integration of the P4HB scaffold and PP meshes was evaluated from SEM images. Host response to P4HB and PP was evaluated by histology and immunohistochemistry (IHC) staining and scoring (for details please see Supplementary material, Table S1). The immune response to P4HB and PP was evaluated by qualification of the foreign body giant cells (FBGCs), polymorphonuclear (PMN) cells, and blood vessels by scoring hematoxylin and eosin (H&E) sections. Masson Trichrome and Verhoef staining were used to visualize and quantify connective tissue, collagen and elastin respectively. IHC staining was performed for the detection of myofibroblasts and smooth muscle cells (α-SMA), macrophage type-1 (M1; HLA-DR) and type-2 (M2; CD163) for indication of fibrotic response and tissue regeneration. The M2/M1 ratio was calculated. Semi-quantitative assessment of all samples was performed by two individual researchers blinded to both time points, using a previously designed grading scale [10, 19].

### Statistical Analysis

Statistical analyses were performed using Minitab19 software (Minitab, State College, PA, USA). Data normality was tested by the Shapiro–Wilk test. Two-way ANOVA was used for normally distributed data and multiple comparisons between individual groups using Tukey’s test. Non-normally distributed data were compared using the Mann–Whitney test. The data are reported as mean ± standard deviation (SD) and as a median with interquartile range (IQR), quartiles 1 and 3, for normal and non-normally distributed data respectively. The significance level was defined as *p* < 0.05.

## Results

### Gross Anatomical Examination of the Explanted Vagina

**Fig. 2:**
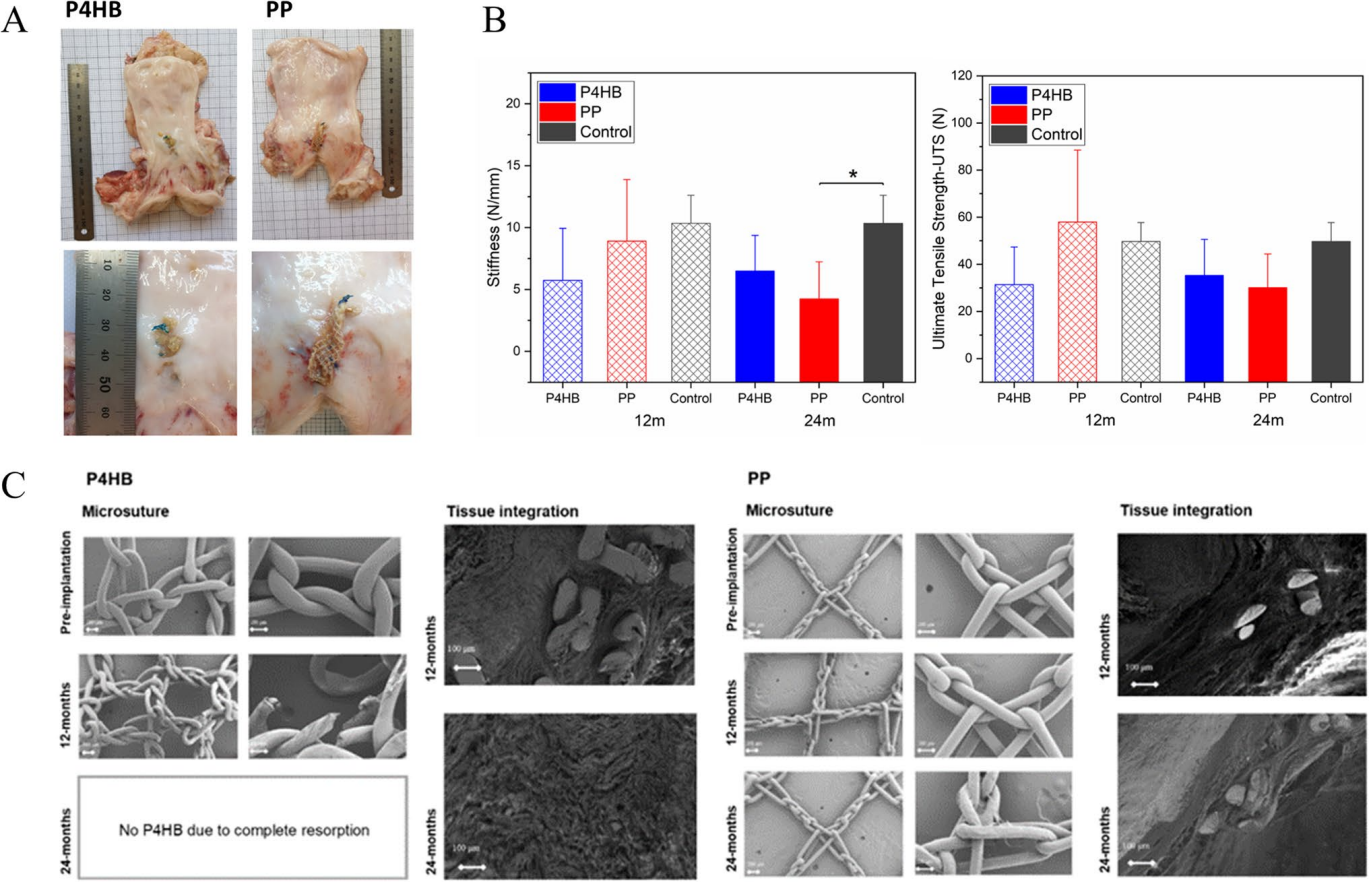
**A** Representative images of vaginal exposures after implantation of the poly-4-hydroxybutyrate (*P4HB*) and polypropylene (*PP*). **B** Stiffness (N/mm) and ultimate tensile strength (*UTS*) of vaginal P4HB and PP explants (*n* = 8 at 12 and 14 months post-implantation) compared with control tissue (tissue harvested from posterior middle vagina; P4HB *n* = 8 and 7; PP *n* = 7 and 8, for 12 and 24 months respectively) at 12 and 24 months post-implantation. *Error bars* represent means ± standard deviations (SD). Two-way ANOVA and multiple comparisons between individual groups using Tukey’s test were used to test for differences between groups and time points. Values differing significantly from the control are indicated by *asterisks*: *p < 0.05. **C** Scanning electron microscopy images illustrating the microstructural properties of P4HB implant and PP mesh following tissue digestion (*left*), and the integration of the P4HB and PP in vaginal tissue at 12 and 24 months post-implantation (*right*)

The surgeries were performed without any complications. There were no surgery-related complications. All animals survived for the complete duration of the study. Both P4HB and PP were well incorporated in the deeper vaginal tissue layers without any signs of encapsulation. No sign of infection or fluid collection was observed in any of the groups. At 12 months post-implantation, small areas of vaginal exposures at the incision site were observed with P4HB scaffold (3 out of 8, sizes of the exposures: 8 mm × 15 mm, 8 mm × 2 mm, and 25 mm × 25 mm) and PP mesh (4 out of 8, sizes of the exposures: 32 mm × 6 mm, 16 mm × 5 mm, one from two locations, each of 3 mm × 5 mm and 30 mm × 30 mm) groups respectively. At 24 months, there was no exposure observed in the P4HB scaffold group, whereas PP mesh had four exposures: 14 mm × 2 mm, 20 mm × 1 mm, 30 mm × 25 mm, and 10 mm × 1 mm. Figure 2A gives representative images of the exposures.

### Passive Biomechanical Properties

Biomechanical properties of the vaginal P4HB and PP explants, and control tissue were compared at 12 and 24 months post-implantation (Fig. 2B). Although not statistically significant, the P4HB explants at 12 months exhibited lower strength and stiffness than PP explants (5.7 ± 5.0 N/mm SD vs 8.9 ± 5.4 N/mm SD) and the control vaginal tissue (10.3 ± 5.9 N/mm SD). At 24 months post-implantation, the stiffness (6.5 ± 3.5 N/mm SD) and the strength of the P4HB explants did not change significantly compared with 12 months post-implantation or the control tissue, even though the P4HB scaffold had undergone nearly complete degradation. The stiffness and strength of the PP explants, however, decreased over time and at 24 months, stiffness (4.2 ± 3.6 N/mm SD, *p* = 0.42) was statistically significantly lower than the control tissue (*p* = 0.04).

### In Vivo Degradation of the P4HB Scaffold

The degradation of the P4HB scaffold was determined by the change in molecular weight (Mw) over time. The average Mw (225.14 ± 3 kDa SD) of the P4HB scaffold declined to 38.5 ± 2.3 kDa at 12 months and was fully degraded at 24 months. P4HB maintained its integrity after 12 months post-implantation and showed good integration within the submucosal layer of the vaginal wall. Surface deformation by means of cracks and fissures on the P4HB fibers and fiber breaks were observed at 12 months via SEM. At 24 months P4HB scaffolds were completely absorbed, which was verified by SEM images. In addition, no residual P4HB material could be obtained from the explants after the tissue removal process. PP mesh remained intact at both time points (Fig. 2C) and were completely integrated with the vaginal tissue at 12 and 24 months post-implantation (Fig. 2C).

### Histomorphology

**Fig. 3:**
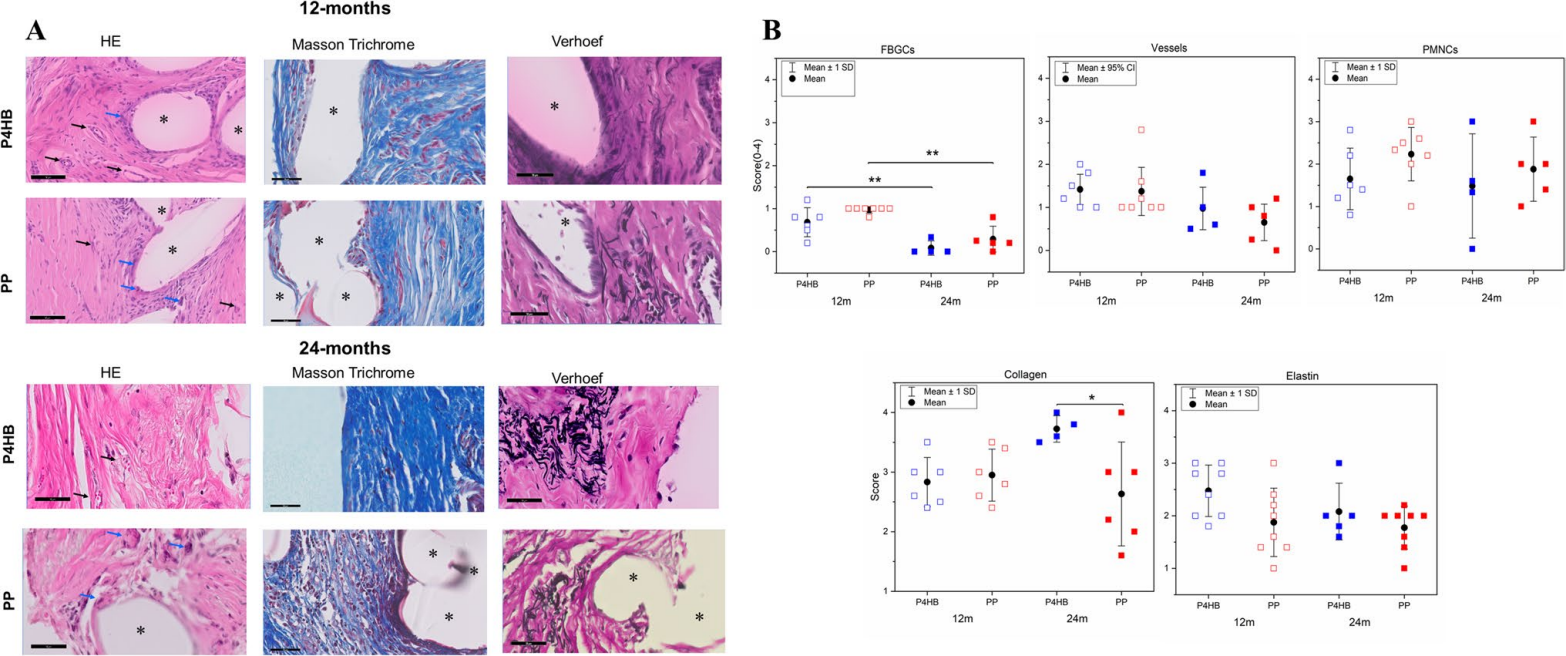
**A** Representative images of H&E, Masson’s trichrome (*MT*), and Verhoef staining of poly-4-hydroxybutyrate (*P4HB*) and polypropylene (*PP*) vaginal explants used for scoring (scale bar, 50 μm). Implant structures are indicated by *asterisks*, foreign body giant cells (*FBGCs*), and vessels are indicated with *blue* and *black arrows* respectively. MT staining: collagen is stained *blue*, cell nuclei are stained *black*, and the background is stained *red*. Elastin fibers are stained *black* in Verhoef staining. **B** Host response to vaginal P4HB and PP implants based on H&E, MT, and Verhoef staining, in terms of FBGCs, polymorphonuclear cells (*PMNCs*), blood vessels, collagen and elastin content at 12 and 24 months post-implantation. (For H&E: P4HB *n* = 7 and 5; PP: *n* = 7 and 5, and for MT and Verhoef: P4HB *n* = 8 and 5; PP: *n* = 8 and 8, for 12 and 24 months respectively). *Error bars* represent means ± standard deviations (*SD*). Two-way ANOVA and multiple comparisons between individual groups using a Tukey’s test were used to test for differences between groups and time points. Values differing significantly from the control are indicated by *asterisks*: *p < 0.05

The P4HB and PP could be identified in between the lamina propria and muscularis layer of the vaginal wall in the H&E-stained samples at 12 months. At 24 months only PP could be identified owing to the complete absorption of P4HB (Fig. S1). At 12 and 24 months, there were statistically no significant differences between P4HB and PP in terms of the presence of FBGCs. There were statistically significantly fewer FBGCs around both P4HB (12 m vs 24 m: 0.7 ± 0.3 SD and 0.1 ± 0.12 SD, *p* = 0.001) and PP (12 m vs 24 m: 1 ± 0.1 SD and 0.2 ± 0.3, *p* = 0.001) at 24 months than 12 months post-implantation (Fig. 3). There was higher inflammatory cell (PMNC) infiltration around the PP mesh than in the P4HB scaffold group at both time points (P4HB: 1.6 ± 0.7 SD and 1.5 ± 1.1 SD; PP: 2.2 ± 0.6 SD and 1.9 ± 0.8 SD at 12 and 24 months). The presence of PMNCs decreased over time for both P4HB and PP. However, the differences in the presence of PMNCs around the P4HB and PP were not statistically significant (Fig. 3B). The mild presence of vessels was observed around the P4HB and PP (Fig. 3) and there were no statistically significant differences in the scores of vessels that are present at the tissue-implant interface for P4HB and PP at both time points. At 12 months, a large presence of mature collagen, which was indicated with darker blue staining, was observed around P4HB (2.8 ± 0.4 SD) and PP (3 ± 0.4 SD). There were no statistically significant differences in the quantity of collagen of the P4HB and PP explants at 12 months. Collagen deposition and maturation around P4HB were increased over time contrary to the collagen around PP. At 24 months post-implantation, the amount of collagen around P4HB (3.8 ± 0.2 SD) was significantly higher than that around the PP meshes (2.6 ± 0.9; P4HB vs PP, *p* = 0.04). Elastin fibers were abundantly present around both implants at each time point and no statistically significant difference in elastin deposition around the P4HB and PP implants was observed at both 12 and 24 months post-implantation (Fig. 3).

**Fig. 4:**
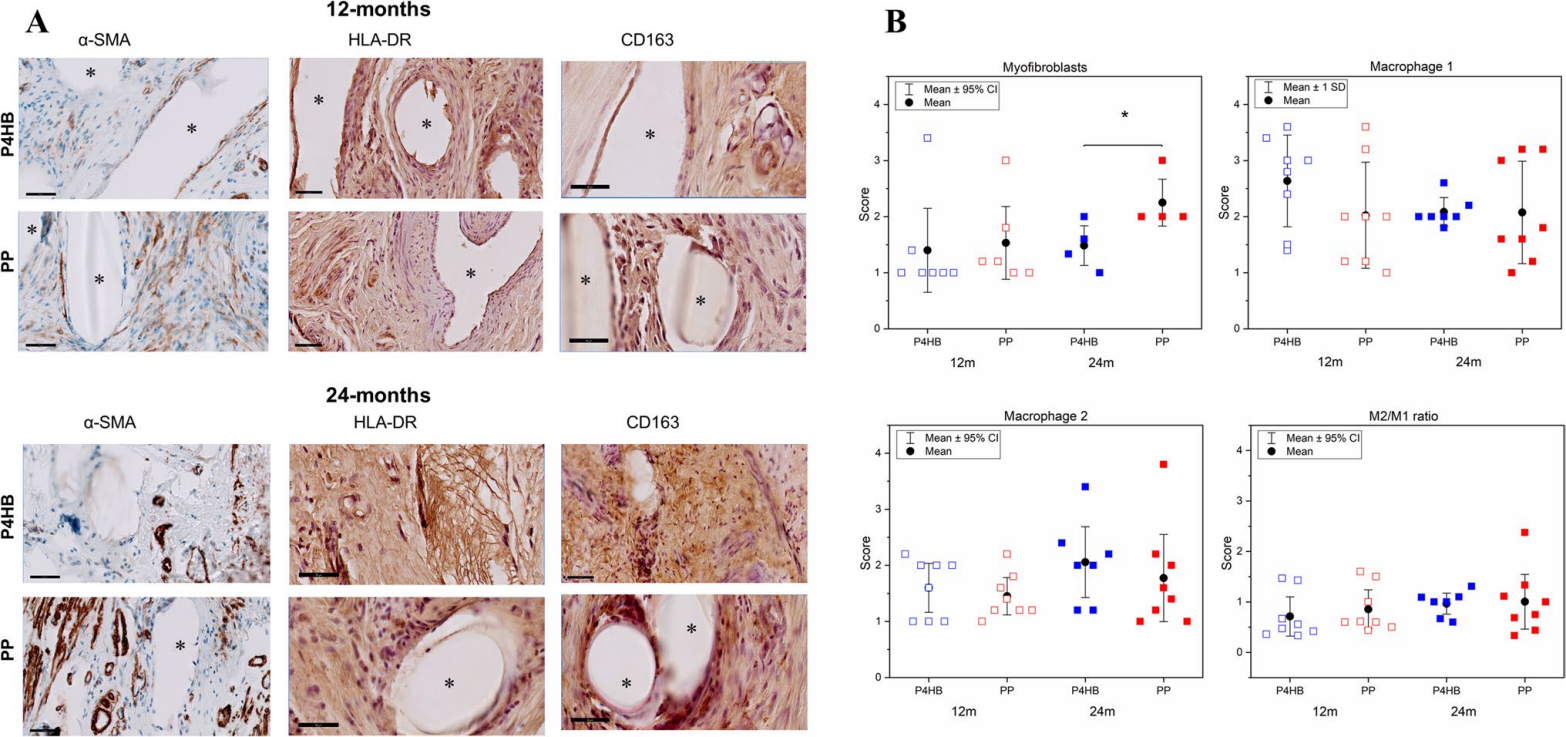
**A** Representative images of immunostained samples at 12 and 24 months post-implantation. Implant structures are represented by *asterisks*. **B** Scoring results for myofibroblast and smooth muscle cells (*α-SMA*), macrophage type 1 (*HLA-DR*), and macrophage type 2 (*CD163*) of poly-4-hydroxybutyrate (*P4HB*) and polypropylene (*PP*) vaginal explants at 12 and 24 months post-implantation. (For CD163 and HLA-DR: P4HB *n* = 7 and 6; PP: *n* = 8 and 8; and for α-SMA: P4HB *p* = 7 and 5; PP: *p* = 6 and 5, at 12 and 24 months respectively). *Error bars* represent means ± standard deviations (*SD*) or median with interquartile range (*IQR*), quartiles 1 and 3, for normal and non-normally distributed data. Two-way ANOVA and multiple comparisons between individual groups using Tukey’s test were used to test for differences between groups and time points. Non-normally distributed data were compared using the Mann–Whitney test. Values differing significantly from the control are indicated with *asterisks*: *p < 0.05

There was mild myofibroblast differentiation around P4HB scaffolds at both time points. Myofibroblast differentiation increased over time after the implantation of PP. There was no statistically significant difference in myofibroblast differentiation around P4HB and PP at 12 months post-implantation. However, P4HB scaffold exhibited statistically significantly lower myofibroblast differentiation than PP (P4HB: 1.3 [1.0–1.8] vs PP: 2.0 [2.0–2.8], *p* = 0.04) at 24 months (Fig. 4).

There was a large presence of macrophage type-1 (M1) and type-2 (M2) around the P4HB and PP implants at both 12 and 24 months post-implantation (Fig. 4). The presence of M2 at the interface of vaginal tissue and P4HB or PP increased over time. However, there were no statistically significant differences in the M1 and M2 scores of the P4HB and PP explants at the two time points. Although there was a slight increase in the M2/M1 ratio of P4HB and PP over time, there was no statistically significant difference in the M2/M1 ratio between P4HB and PP (Fig. 4).

## Discussion

In this study, we performed long-term preclinical evaluation of a prototype absorbable vaginal P4HB scaffold compared with a nondegradable PP mesh. The stiffness of the vaginal P4HB explants was almost unchanged, even though the P4HB underwent significant degradation over time, leading to complete absorption at 24 months post-implantation. On the contrary, the stiffness of the vaginal PP explants decreased between time points, reaching significantly lower stiffness than the control tissue at 24 months. The collagen amount in the vaginal tissue after P4HB implantation was significantly increased over time, whereas it remained the same for the PP mesh. In addition, no exposures were observed in the P4HB group, unlike the PP group, at 24 months.

The P4HB degrades gradually over time, allowing for new tissue regeneration and growth [4, 10, 22]. This is in contrast to other degradable materials that lose mass more quickly, resulting in weak tissue and insufficient load-bearing capacity, which may lead to disappointing surgical outcomes such as recurrence [23, 24]. Synthetic polyglactin-910 completely absorbed after only 6 weeks post-vaginal implantation in patients and caused cystocele recurrence (25%) at 12 months [23]. Studies on resorbable methoxy polyethylene glycol-polylactic-co-glycolic acid polymer [24] or electrospun ureidopyrimidinone-polycarbonate in POP [19] have also raised concerns regarding their rapid degradation and poor surgical outcomes. Clinical studies of xenografts likewise indicate higher recurrence rates owing to the fast degradation of the natural implants [25, 26]. P4HB, with the advantage of prolonged degradation, resulted in a restoration of strong tissue, which was evidenced by the improved mechanical properties of the P4HB-implanted tissue that was shown by maintenance of stiffness and strength over time. On the contrary, the stiffness and strength of the PP explants declined over time and were significantly lower than in control tissue at 24 months. The explant stiffness results from the combination of the stiffness of the implant, the underlying grafted vagina, and newly ingrown tissue [6]. We previously showed that the P4HB and PP implants exhibit comparable stiffness values before implantation [4]. Considering that P4HB was fully degraded at 24 months, its contribution to the stiffness of the explant can be assumed to be limited if not zero. Therefore, the maintenance of stiffness of P4HB explants is worth observing, as well as the supporting effect of the P4HB results from the remodeled ingrown tissue.

No infections, fluid collections, or synechiae were observed after the implantations. Exposures on P4HB and PP were observed in both groups at 12 months post-implantation, but only for PP mesh at 24 months. Exposures in the PP group might be attributed to the higher membrane stiffness [10] and the high region stiffness [4] values of PP mesh compared with P4HB before implantation. There is a higher risk of exposure after using stiffer meshes, as they can create stress shielding in the vaginal wall and cause thinning of the underlying vagina, which results in mesh exposures and erosions [8]. Even though the sheep is a highly translatable model for the host response and biomechanics and gives a good indication of clinical complications, it should be noted that this study is not powered to evaluate complications. In our previous short-term study, we did not observe any exposures after vaginal implantation of P4HB [10]. De Tayrac et al. observed a 33.3% exposure rate with PP mesh after vaginal implantation into sheep at 12 weeks post-implantation [20]. Another study reported 50% and 16.7% vaginal PP exposures after 60 and 180 days respectively [15]. Degradable implants have the advantage of low erosion and exposure rates. No erosion or exposure has been reported for the degradable xenografts such as InteXEN® [26] and Surgisis® [25]. A randomized controlled trial [27] reported one case of mesh erosion after a median follow-up of 23 months in women who had anterior repair with polyglactin 910. Exposures occur mostly in the long term [28] and our data suggest that P4HB might eliminate the long-term adverse events-exposures. The exposure rates might be higher than what is observed clinically. Indeed, our systematic review showed that exposure rates in animal studies are higher than those in women, which might be explained by the different vaginal environment, the experience of the surgeon in the technique, and possibly the use of more experimental types of implants in animal studies [29]. In addition, meta-analyses of this review showed that the exposures are more common in larger animals than in small animals. Larger implants were used in large animals (sheep and rabbits); these cause a greater mesh burden and have been shown to be a risk factor for vaginal exposures, which is in line with observations in women [16, 30]. Both P4HB scaffold and PP mesh exhibited mild inflammatory responses that declined over time. Mild immune response emphasizes the compatibility of the material with the vaginal tissue, indicating in human cases no or minimal clinical complications. The inflammatory cells can influence fibroblast behavior, extracellular matrix deposition, tissue regeneration, and healing [31, 32]. At 24 months, there were almost no FBGCs owing to the complete degradation of the P4HB; instead, well-remodeled and mature connective tissue replaced the scaffold. Collagen levels in vaginal tissue significantly increased over time with P4HB implantation, while remaining the same with PP mesh. This suggests that collagen deposition contributes to maintaining vaginal tissue stiffness with P4HB explants [6, 10]. More collagen and elastin indicate that mechanically stronger vaginal tissue might therefore translate into successful surgical outcomes in human cases. Additionally, tissue remodeling and regeneration were superior with P4HB implantation to PP implantation, as shown by the decrease in myofibroblasts in P4HB explants over time while remaining the same in PP explants. During the tissue remodeling phase; collagen matures and when the tissue is sufficiently remodeled and repaired, α-SMA expression becomes down-regulated, which results in a decreased number of myofibroblasts [2, 33]. An increased number of myofibroblasts is associated with adverse events such as fibrosis or exposure [2, 10, 34] and the high presence of myofibroblasts after 24 months in the PP explants may be associated with impaired healing. This may suggest that P4HB might be less prone to clinical complications such as exposure or fibrosis in women than PP.

No significant differences were found in the M2/M1 responses of P4HB and PP. A higher M2/M1 ratio indicates proper healing and tissue regeneration, which means that the implant results in improved tissue quality after surgery; therefore, the POP tissue regains its mechanical properties and functionality. In our previous short-term study, we found a higher M2/M1 ratio on the P4HB explants than on the PP [10]. M1-like macrophages are pro-inflammatory and responsible for inflammatory signaling in the early phases of the healing process, whereas M2-like macrophages contribute to tissue healing and regeneration in the resolution phase. In the case of proper tissue healing, an inflammatory response declines over time [2, 35]. P4HB scaffolds promote tissue regeneration even in the long term, providing a remarkable and desired host response.

The P4HB scaffold that is used in this study is not the final, commercial medical device; therefore it is challenging to draw firm conclusions on the cost effects or savings associated with P4HB. However, studies showed that P4HB resulted in improved clinical outcomes with fewer recurrence rates and complications for hernia repair and resulted in cost savings compared with biological tissue-based degradable materials [36, 37]. Phasix (P4HB) mesh turned out to be cost-effective in hernia repair in the long term compared with PP. Even though P4HB was associated with increased initial costs, abdominal morbidity is significantly decreased after follow-up beyond 2 years [38]. When available, cost-effectiveness analyses of P4HB scaffold should be conducted in clinical studies on POP with short- and long-term follow-up. These analyses should include complications, recurrences, and reoperations for prolapse [39].

We acknowledge the limitations of this study. The sheep used in this study are not inbred animals and therefore an in-depth investigation of the immune response is not possible. The outcomes of the study, especially the gross anatomical outcomes such as exposures, can be affected by external factors such as the learning curve of the surgeon, and animal species used in the study. For instance, in our previous sheep study, we did not observe any exposures in P4HB and PP (retrospective data) group at 60 and 180 days [10]. Even though this may be related to the duration of the studies, it should still be noted that the surgeries were performed by different surgeons and the sheep species used were different. Although the vaginal sheep model offers insight into host response and in vivo degradation, the forces applied to the vagina and pelvic floor in sheep and humans differ because of differences in standing position, as humans are bipedal, and sheep are standing on all fours.

On the other hand, this study, to the best of our knowledge, is the longest preclinical evaluation of absorbable vaginal scaffolds. In addition, this study is the first long-term preclinical comparison of a degradable scaffold with permanent PP mesh for POP. The duration of the study allowed us to demonstrate the long-term performance of the P4HB scaffold and the vaginal tissue properties after the complete resorption of the implant. The prototype P4HB scaffold used in this study has been thoroughly characterized [4] and its short-term host response [10] was demonstrated in our previous studies. Also, we used a well-established animal model used in pelvic floor research [10, 15, 18, 19].

Even so, clinical trials in humans are needed to further evaluate the safety and efficacy of the P4HB scaffold in POP treatment. Still, after thorough preclinical studies, the P4HB scaffold appears very promising as a treatment for POP and may provide favorable risk–benefit to patients seeking optimal treatment options for POP.

## Conclusions

The P4HB scaffold provided excellent support to the vaginal tissue and resulted in a mechanically self-sufficient tissue after complete degradation. Unlike PP mesh, which causes a decline in tissue properties, the P4HB scaffold is a long-lasting, absorbable synthetic implant that can benefit patients needing pelvic floor reinforcement for the repair of POP.

### Supplementary information

Below is the link to the electronic supplementary material.

Supplementary file1 (DOCX 1036 KB)

## Data Availability

Data are available upon request.
