# Supplementary material for: Two-Year Preclinical Evaluation of Long-Term Absorbable Poly-4-hydroxybutyrate Scaffold for Surgical Correction of Pelvic Organ Prolapse
Source: Int Urogynecol J. 2024 Mar 2;35(3):713–22. doi: 10.1007/s00192-023-05720-0 (PMC11024044; doi:10.1007/s00192-023-05720-0)
Supplement: Supplementary file 1 — Supplementary file1 (DOCX 1036 KB) [file 192_2023_5720_MOESM1_ESM.docx]

**Supplementary material 1: Surgical procedure**

All animals underwent vaginal wall reconstruction with the implant under sterile conditions. Premedication (30min.) and antibiotic prophylaxis (10min.) was administered prior to induction of anaesthesia by insertion of intravenous catheter into the cephalic vein and Midazolam (0.3 mg/kg) and Buprenorphine (0.01 mg/kg) are administered. Anaesthesia was induced with IV Ketamine (3 mg/kg) injection, following intubation and maintained with 2% Isoflurane in 5 L/min oxygen. Intravenous fluid (Ringers Lactate) was given via the cephalic vein during surgery. Prophylactic antibiotic IM (Excenel RTU, 1mg/kg) was administered intra-operatively till 2 days postoperative. Sheep were placed in the dorsal decubitus position with pelvic limbs secured in hip flexion to allow vaginal implantation. The urinary bladder and rectum were emptied, surgical areas were sheared. Vagina and perineum were disinfected with Polyvidone Iodium 7.5% (Braunol^®^, B. Braun Medical, Belgium) and covered by a sterile drape.

Retractor and hooks (Lone star^®^ retractor system; Trumbull, USA) were placed to make wide access to the vestibulum vaginae. The posterior vaginal wall was grasped with Allis forceps 3 cm cranially from the hymen. Aqua-dissection with 10 ml Hartman solution was performed in the posterior vaginal wall 2 cm cranially to the hymeneal ring, then a longitudinal 3 cm incision was made at that place. Hooks were used to open incisions. The rectovaginal septum was bluntly (finger and gauze) and sharply (scissors) dissected to create a suitable space for implant between the vaginal epithelium and rectal serosa. Implants were fixed with interrupted 3/0 polypropylene sutures (Prolene^®^, Ethicon, Zaventem, Belgium) firstly in the corners and then halfway along its borders. The vaginal wall was closed with a running 3/0 polyglactin 910 (Vicryl) suture. A vaginal tampon was inserted for 24 hours, and sheep were examined for postoperative complications. Animals were checked for postoperative complications and housed until one week after surgery at the animal facility and then at the farm with unrestricted access to food, water, and chew with free access to open space. Sheep were euthanised at 12- and 24- months after surgery.

Postoperative analgesia contained Ketofen 10% (Ketoprofen 0.1 gr/ml) 3ml / 100kg was administered intramuscular into the gluteal muscle for 3 consecutive days after surgery. Animals were clinically observed for one week. Surgical sites were regularly examined to note early postoperative complications. Sheep were euthanised by injection of 50 ml of Sodium Pentobarbitone (200mg/ml) via the jugular or cephalic vein at 12- or 24 months after surgery.

**Supplementary material 2: Animals used and ethics**

Animals were treated in accordance with current national guidelines on animal welfare. The sample size was calculated using a two-sided Fisher exact test with 5% significance level and 90% power to detect the 50% expected differences between the explant and control stiffnesses of comfort zone (N/mm) as quantitative endpoint. Sheep were seven years old having had each more than four vaginal deliveries and weighing 51.5 ± 5.7 kg. None of the ewes had obvious prolapse, however formal staging of the prolapse was not performed. Animals were housed 5 days after surgery at the animal facility and then at the farm with unrestricted access to the food, water, chew and with free access to open space.

**Supplementary material 3: Blinding**

During the study, investigators and caregivers were blinded from knowledge of which intervention each animal received. An identification number was randomly given to each animal and the surgeons were not informed about the identification numbers and about the corresponding treatment groups (for implants and time points). All animals were operated and examined in a random order during the entire study. Outcome measurements and data analysis were performed by two individual researchers blinded by implant groups and time points (only for histology and IHC).

**Supplementary material 4: In vivo degradation of P4HB**

In vivo degradation of P4HB implant were determined by molecular weight (Mw) change via gel permeation chromatography (GPC) analysis. The changes in morphology of P4HB and PP meshes were monitored via scanning electron microscopy (SEM). Both GPC and SEM analyses were performed following to removal of the vaginal tissue by digestion.

**Vaginal tissue digestion:** Vaginal P4HB and PP explants were incubated overnight with collagenase (type I, from Clostridium histolyticum) solution (1.0mg/mL) in TESCA buffer (50mM TES, 2mM CaCl2, 10mM NaN3, pH 7.4) at 37°C shaker (600 rpm/min) and were then rinsed with sterile water, 70% ethanol and air dried.

**Gel permeation chromatography (GPC):** P4HB implant samples were dissolved at 1mg/mL in chloroform, filtered using a 0.45 𝜇m filter to remove undigested particulates, and 95 𝜇L of this solution was then injected onto a GPC column. GPC was performed in chloroform at 1ml/min using a Polymer Labs, PLgel column (5micron, mixed C, 300 × 7.5 mm) with an Agilent 1100 Series HPLC with RI detector. Calibration was conducted against monodisperse polystyrene standards. Molecular weight is reported in units of Daltons (Da).

**Scanning electron microscopy (SEM):** After tissue removal, P4HB and PP implants were coated with a layer of gold about 3 nm thick in an EMITECH K650X sputter coater and examined at room temperature in a JEOL JSM6700F field emission scanning electron microscope at an accelerating voltage of 2.5 kV.

**Supplementary material 5: Histology**

Staining was performed on 5 µm sections and scoring of histology and immunohistochemistry (IHC) slides were performed by two researchers blinded to the groups and the time points on five randomly chosen non- overlapping fields per slide (5 samples per group) were scored at a magnification of ×400 and averaged (n=5). Fields were randomly selected at the interface between the implant and surrounding tissue. In case of any disagreement on scoring, a senior researcher was consulted for a third opinion.

The P4HB implant and PP mesh could be identified in between the lamina propria and muscularis layer of the vaginal wall in the Hematoxylin and Eosin (H&E) stained samples. HE stains were performed to quantify the presence of foreign body giant cells (FBGC), polymorphonuclear (PMN) and vessels. An ordinal scale was used similar to that described in our short-term study [[1](#_ENREF_1)], where scores are made as follows: none of the cells/vessels per high-power field (score 0), 1–5 (score 1), 6–10 (score 2) and >10 (score 3). Masson’s trichrome stains extracellular connective tissue (mainly unspecified collagen) blue. Five non-overlapping images 400x magnification were obtained and semi-quantitatively evaluated for collagen at interface mesh-surrounding tissue. Verhoef stains the elastin fibers black. Samples were scored from 1-4 (Score 1: mild presenced Score 2: large presence Score 3: abundance and Score 4: great abundance.

For IHC staining, sections were processed with a mouse and rabbit specific HRP/DAB (ABC) Detection IHC Kit (Abcam) and three different monoclonal antibodies (Table S1). Antibodies were diluted in 1% bovine albumin serum (BSA) (Sigma-Aldrich). Semi-quantitative assessment of the extent of immunostaining was performed using a qualitative grading scale; absent=0, mild presence=1, large presence=2, abundance=3, great abundance=4. Example photographs (Figure S1) depicting 1, 2, 3 and 4 were provided for reference. The M2/M1 ratio was also calculated for each group using the values from the blind scoring of the immunostaining (n=5). For calculation of macrophages M2/M1 ratio were used to determined scores. On apha-SMA images, smooth muscle and vessels were excluded from evaluation.


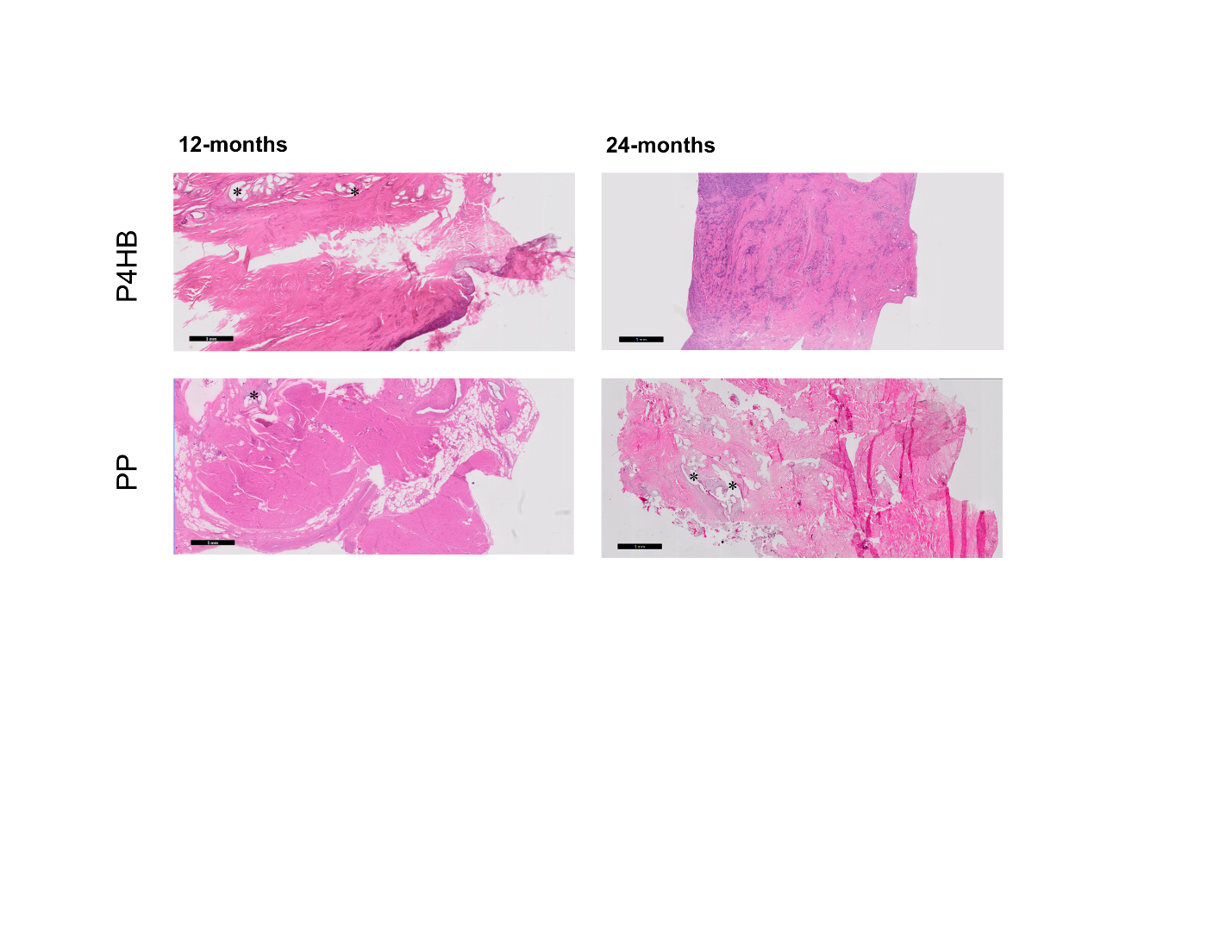


**Figure S1:** HE staining indicating that the P4HB implant and PP mesh could be identified in between the lamina propria and muscularis layer of the vaginal wall.

**Table S1:** Table contains used antibodies, their concentrations and companies of their origin: Abcam (Cambridge, UK), DAKO (Glostrup, Denmark), AbD Serotec (Kidlington, UK)

| Antibody | Concentration | Company |
| --- | --- | --- |
| mouse anti-smooth muscle actin (SMA) | 1:200 | DAKO |
| mouse anti-HLA-DR | 1:100 | Abcam |
| mouse anti-CD163 | 1:200 | AbD Serotec |

**REFERENCES**

1. Diedrich, C.M., et al., *Evaluation of the short‐term host response and biomechanics of an absorbable poly‐4‐hydroxybutyrate scaffold in a sheep model following vaginal implantation.* BJOG: An International Journal of Obstetrics & Gynaecology, 2021.
